# Development and Application of a Mind Map to Improve the Adolescent Behaviour on Mental Health and Substance Abuse in Rural Odisha: A Community-Based Quasi-experimental Study Design

**DOI:** 10.7759/cureus.93981

**Published:** 2025-10-06

**Authors:** Payel Roy, Abhisek Mishra, Arvind K Singh, Swayam Pragyan Parida, Nisha Murmu, Asmita Patnaik

**Affiliations:** 1 Community Medicine and Family Medicine, All India Institute of Medical Sciences, Bhubaneswar, Bhubaneswar, IND; 2 Community Medicine, Employees' State Insurance (ESI) Medical College, Bangalore, Bangalore, IND

**Keywords:** adolescent addiction, adolescents, mental health, mind mapping, substance abuse

## Abstract

Introduction

Adolescents in India face significant health challenges, particularly mental health issues and substance abuse. With a large proportion of the population under 18 years, addressing these concerns is crucial. Substance abuse and mental health issues often begin in adolescence due to peer pressure and socio-economic factors, leading to long-term health problems.

Objective

This study aims to evaluate the effectiveness of mind/concept maps in enhancing adolescent mental health and reducing substance abuse in a rural community in Khordha, Odisha, India.

Methodology

This community-based quasi-experimental study was conducted from January to July 2024 in the rural block of Tangi, Khordha. Adolescents aged 18-19 years attending the Community Health Centre's (CHC) outpatient department (OPD) were included in the study. A mind map tool was developed from qualitative interviews exploring themes related to mental health and substance abuse. A total of 22 adolescents were interviewed for tool development, and 38 adolescents completed pre- and post-intervention assessments using the Patient Health Questionnaire-9 (PHQ-9) (for mental health) and the World Health Organization's (WHO) Alcohol, Smoking, and Substance Involvement Screening Test (ASSIST) tools. Data were analysed using paired t-tests, and qualitative themes were also explored.

Results

The pre-test mean PHQ-9 score among the participants was 7.06 (SD=3.38), which decreased to 5.78 (SD=2.71) post-intervention, showing a significant reduction (p < 0.001). Similarly, WHO ASSIST scores reduced from 2.89 (SD=4.62) to 1.83 (SD=3.55), with a significant correlation (p < 0.001). Qualitative data revealed that stress, peer influence, and family issues were major factors in substance use and mental health problems.

Conclusion

The application of mind/concept maps significantly improved mental health outcomes and reduced substance abuse among adolescents. This intervention offers a promising approach to address these issues in rural settings and could be expanded to other communities to enhance adolescent health and well-being.

## Introduction

Globally, India has one of the highest proportions of children and adolescents, with 45% of the population aged under 18 years and 35.3% within the 5-19 years age group [1]. The major health issues faced by adolescents include mental health problems, early pregnancy and childbirth, HIV/sexually transmitted infections (STIs) and other infectious diseases, violence, unintentional injuries, malnutrition, and substance abuse [2]. Adolescents often become exposed to addictive substances like tobacco, alcohol, and psychotropic drugs such as cannabis, cocaine, and heroin due to peer influence or social stress [3]. A multicentric cross-sectional study across 15 states in India has found that the prevalence of tobacco use is 26.4%, alcohol 26.1%, and cannabis 9.5% [4]. Approximately 40% of first-time smokers are under 18 years old, and annually, about 55,000 children in India, primarily from low socio-economic backgrounds, take up smoking [5]. This risky behaviour is often initiated during childhood and adolescence, as over 70% of adult smokers report that they started smoking daily before adulthood [5].

Globally, one in seven individuals aged 10-19 years experiences a mental disorder, accounting for 13% of the global disease burden in this age group [6]. Depression, anxiety, and behavioural disorders are leading causes of illness and disability among adolescents, and suicide is the fourth leading cause of death among 15-29-year-olds [6]. According to the National Mental Health Survey 2015-2016, the prevalence of mental disorders in the 13-17 years age group is 7.3%, with an almost equal distribution between girls and boys [7]. Over 50% of mental health disorders begin before the age of 14 years [8].

In the Diagnostic and Statistical Manual of Mental Disorders, Fifth Edition (DSM-5), substance use is not described by a single definition but through the diagnostic criteria for Substance Use Disorder (SUD). SUD is characterised by a problematic pattern of substance use that results in clinically significant impairment or distress. A diagnosis is made when an individual meets two or more of the 11 specified symptoms within a 12-month period (F10) [9]. Substance abuse poses a significant barrier to the survival, protection, growth, and development of healthy children, which is essential for improving quality of life. Substance abuse among youth is uniquely associated with an increased risk of psychiatric disorders such as depressive disorders, anxiety disorders, attention deficit hyperactivity disorders, and conduct disorders [10]. A secondary data analysis by Smith et al. supported the relationship between substance abuse and mental health, suggesting that psychoactive substances are often used for self-medication to alleviate distress symptoms [11]. Early onset of substance abuse is also associated with higher comorbid conditions, a more chronic course, and poorer outcomes [12]. Various factors, including emotional and behavioural problems, family issues, and relationship challenges, can negatively impact adolescent mental health [13].

Government health programs, such as the Rastriya Kishore Swasthya Karyakram (RKSK), address six strategic areas: nutrition, sexual and reproductive health, non-communicable diseases, substance misuse, injuries, violence, and mental health [2]. The Adolescent Reproductive and Sexual Health (ARSH) program in India aims to provide youth-friendly health services yet faces challenges such as cultural stigma, lack of awareness, and limited access to services [2]. While ARSH focuses on reproductive health, it often overlooks comprehensive sexuality education and mental health, leaving gaps in adolescent care [14]. Although adolescent health clinics are operational across India, awareness remains low, with studies reporting that only 9.5% of adolescents are aware of adolescent-friendly health clinic services [14].

Mind mapping is a visual strategy that begins with a central idea placed at the centre of a page, from which related concepts branch outward, forming a structured diagram. It is used to brainstorm, organise thoughts, highlight connections, and simplify complex topics, making it especially useful for note-taking, essay planning, problem-solving, and exam preparation by enhancing creativity, memory, and understanding [15]. Mind mapping offers several advantages over conventional tools, such as cluster diagrams, fishbone diagrams, Venn diagrams, and causal loop diagrams. It helps in focusing on key ideas, mapping knowledge in a structured way, and understanding and retaining information. Mind mapping can facilitate behaviour change by enhancing perceived behavioural control, leading to habit change in line with the theory of planned behaviour (the Theory of Planned Behaviour explains that a person's actions are driven by their intentions, which are shaped by attitudes, social norms, and perceived control over the behaviour) [16,17].

The prevalence of substance abuse is particularly high in Odisha, where 44% of individuals use at least one substance [18]. Furthermore, there is a high burden of mental health disorders in rural areas of Odisha, with 8.8% experiencing mild mood disturbances, 15.2% borderline depression, 12% moderate depression, and 7% severe depression in adolescents [19]. This highlights the urgent need for interventions to improve adolescent mental health and address substance abuse in rural areas of the state. Engaging adolescents to understand and explore their thoughts and perceptions through mind mapping can be an effective strategy.

Although adolescent health clinics are operational across India, awareness of these services remains low. The ARSH program mainly focuses on reproductive health, where it overlooks the mental health and substance use aspects [2]. Although literature is available on various interventions, to date, it has never focused on the brainstorming of adolescents, resulting in behaviour change in them [20]. Mind mapping helps focus on key areas, maps knowledge in a structured way, and facilitates understanding and retention of information. Henceforth, this study aims to investigate the use of mind mapping-based interventions in the self-dealing of mental health and substance abuse disorders among adolescents in the rural community of Odisha, where the literature is already extensive.

## Materials and methods

A community-based, quasi-experimental, prospective study was conducted in the rural block of Tangi, Khordha district, Odisha, from January 2024 to July 2024. This community-based quasi-experimental design has been executed in two phases: the first phase for the development of the mind map, and the second phase of the study aims to assess the impact of the mind map intervention on adolescents.

First part: mind map development

In this prospective study, adolescents aged 18-19 years attending the Community Health Centre (CHC) outpatient department (OPD) were included by convenience sampling for the development of a mind-mapping tool. The study population was selected based on specific inclusion and exclusion criteria. The inclusion criteria required respondents to have used any substance in the past year, to be accompanied by a parent or another guardian at the hospital, and to have sufficient cognitive ability to understand and comprehend the study requirements. Adolescents were excluded if they had any established mental disorder or cognitive disability, as indicated by medical records or by prior consultation with a physician.

**Figure 1:**
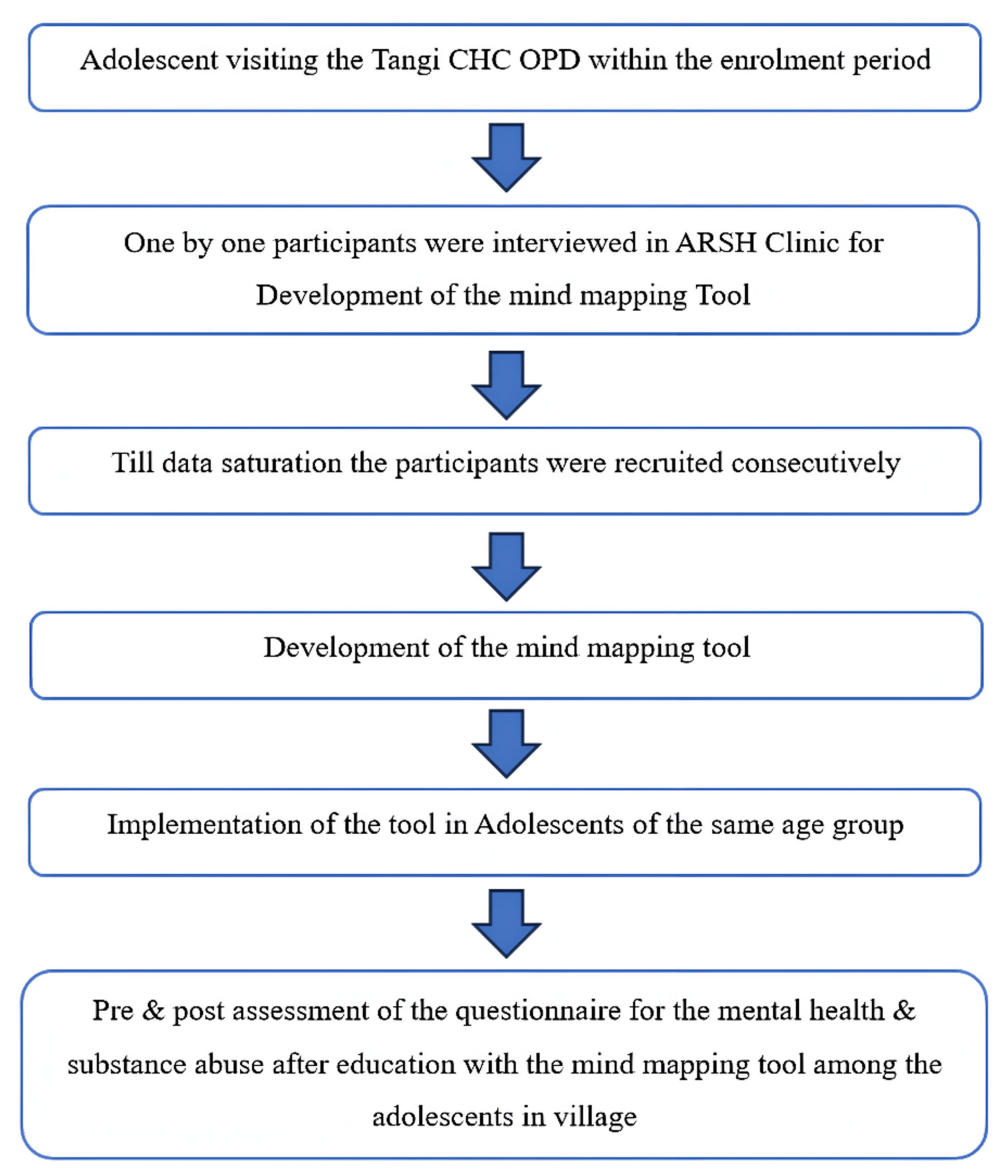
Flow diagram depicting the methodology of the study CHC OPD = Community Health Centre Outpatient Department, ARSH = Adolescent Reproductive Sexual Health

**Figure 2:**
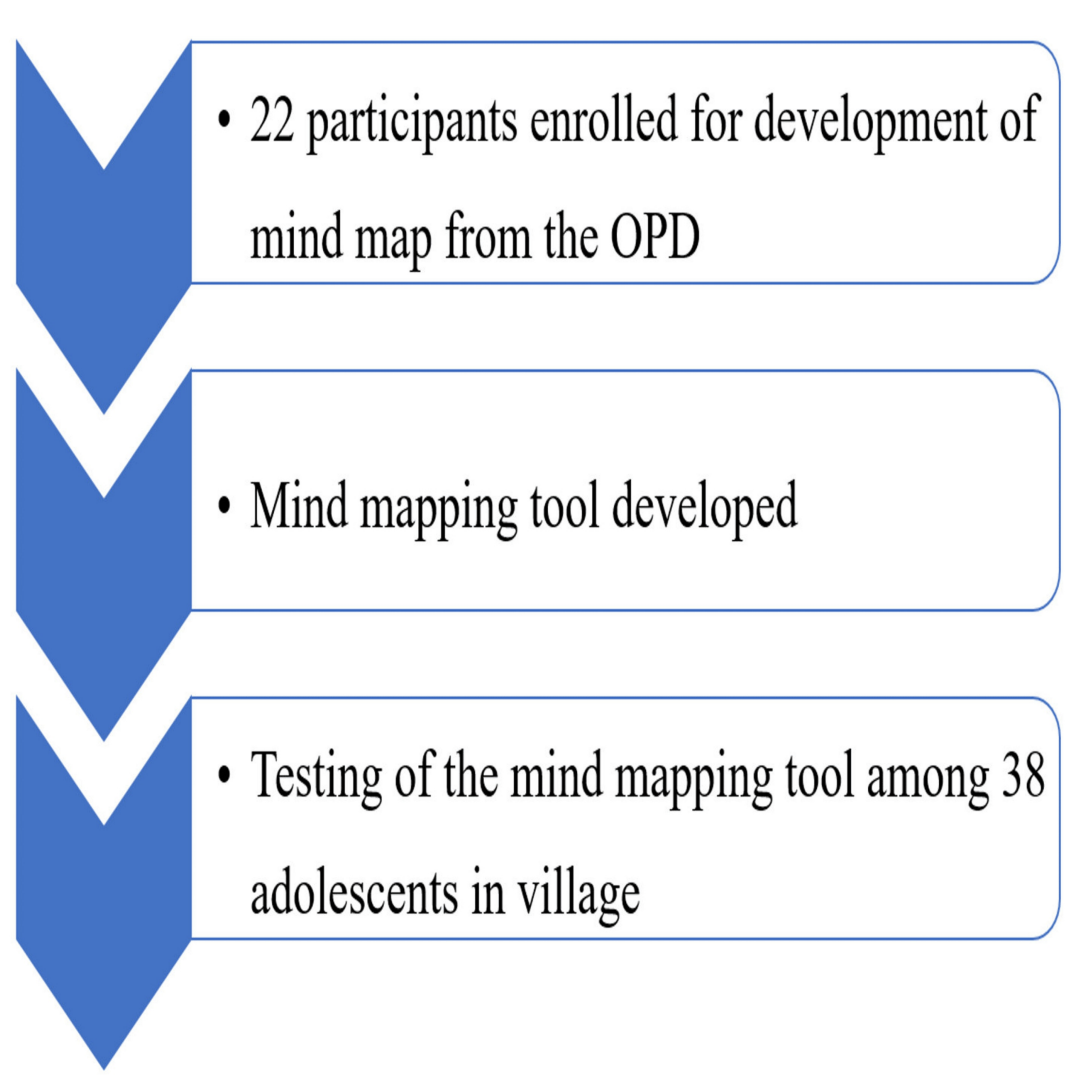
Flow of participants in the study OPD = Outpatient Department

A total of 22 participants, including both genders, were interviewed until data saturation was achieved for the development of the mind mapping tool at the ARSH Clinic of the CHC (Figures 1, 2).

A qualitative, in-depth interview was conducted to develop the mind-mapping tool. The recorded interviews were transcribed verbatim by the first researcher. The accuracy of the transcript was verified by comparing it with recorded files. The transcripts were initially created in the local language and presented to the participants for content validation. In case of any discrepancy, the part was corrected or removed. Then, the transcript was converted into English.

After the in-depth interview, transcripts were shown to participants for verification; four of them declined to proceed with the transcript. Two researchers involved in the study independently coded the transcript. Based on the coding, codebooks were developed. The codes were presented to a third researcher to achieve consensus. Finally, the codes were grouped into categories, themes, and sub-themes by two independent researchers. The themes were finally reviewed, and definitions of each were provided.

A total of 18 participants' transcripts were analysed to develop the mind-mapping tool. The research team consists of four members: one resident doctor, two nursing officers, and one medical social worker. The study subjects received theoretical and practical demonstrations on harmful substance use and its ill effects, as well as on mental health disorders. The patients' queries were thoroughly discussed and resolved. The content of the exercise was summarised, and every participant was provided enough opportunity to raise all of their concerns.

**Figure 3:**
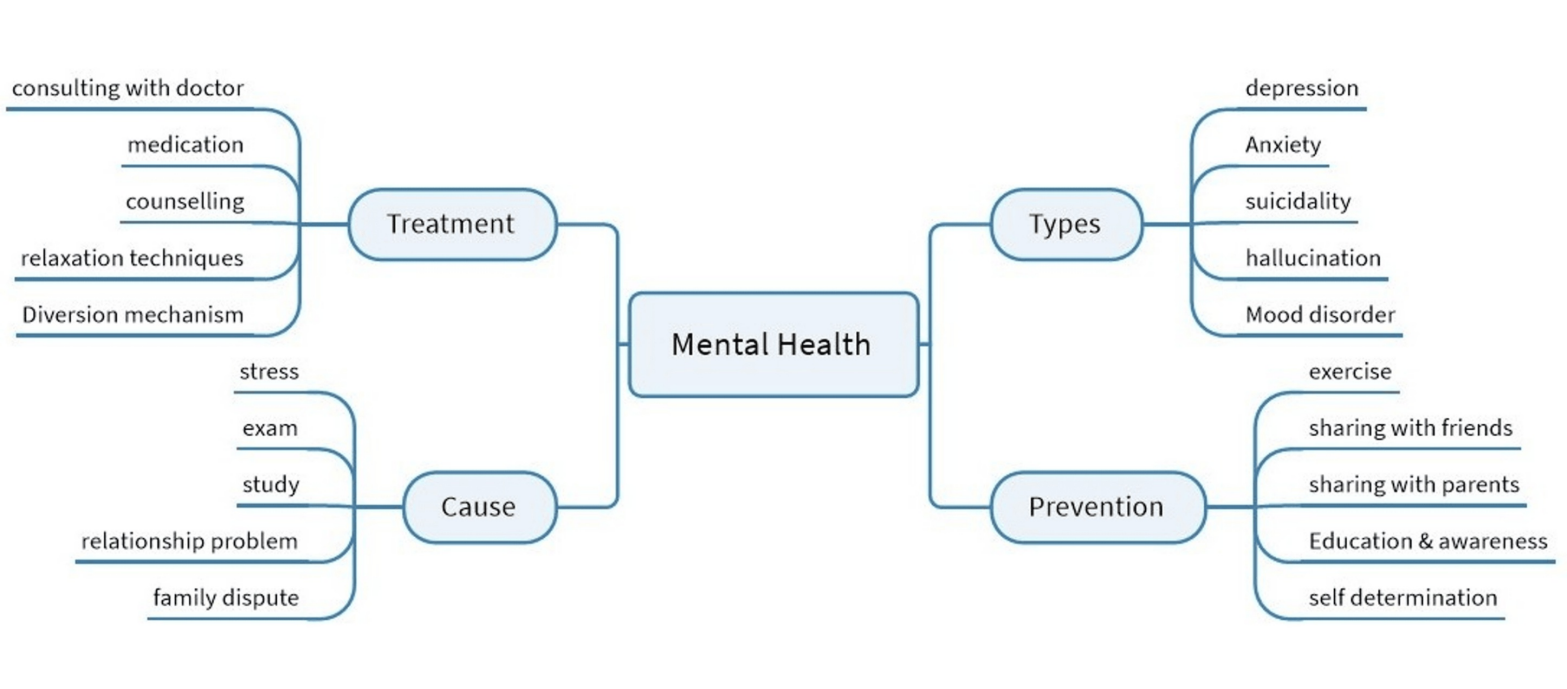
Mind map reflecting perceptions about mental health among adolescents

**Figure 4:**
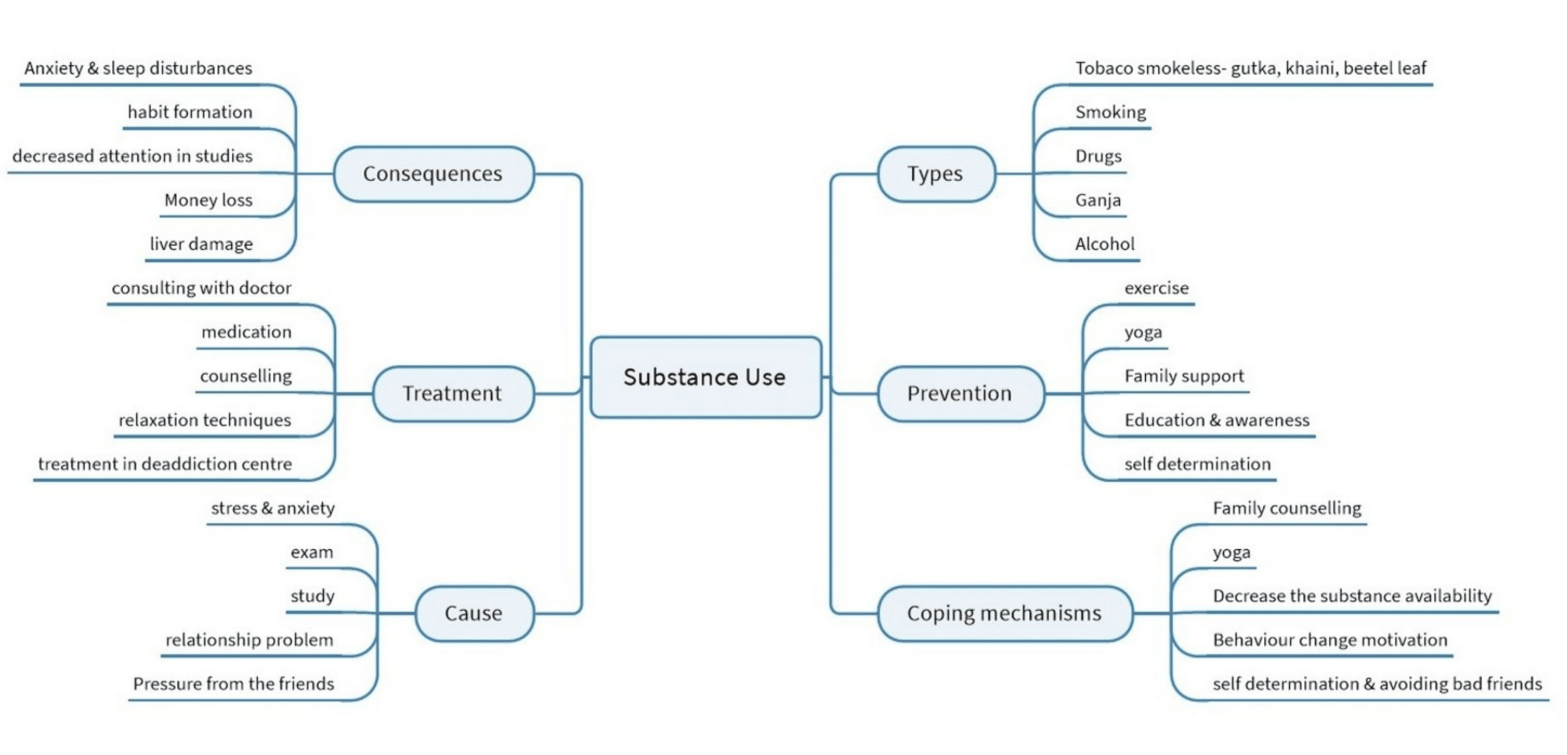
Mind map reflecting perceptions about substance use among adolescents

MindManager software version 23 was used to construct the mind maps by the primary researcher (P), who has prior knowledge in qualitative research. The mind map consists of two modules: one for substance abuse and another for mental health in adolescents. The substance abuse module has six additional branches covering causes, types, treatment, consequences, prevention, and coping mechanisms. The mental health module has four branches for cause assessment, types, treatment options, and prevention. The preliminary mind map was discussed among team members and reviewed by external experts, then converted into the final version, printed, and plastic-encapsulated. The tool developed through in-depth interviews of the adolescents highlights the main theme, domains, and subsequent nodes in each domain (Figures 3, 4).

The trustworthiness of the study was ensured in several ways. Transferability was achieved by providing detailed information about the study setting, sample, and sampling methods, as well as the participants' demographics and the procedures used for conducting the interviews. To confirm the study's findings were reliable or dependable, the data were analysed using Braun and Clarke's method, which is a well-established approach for analysing data (Braun and Clarke's method is a widely used approach for thematic analysis, which follows six phases: familiarisation, coding, generating themes, reviewing themes, defining and naming themes, and producing the report) [21]. To ensure personal opinions did not influence the findings, a codebook was created after reviewing the first three interview transcripts. Two researchers agreed on the codes to be used for the remaining interviews and also met to determine the main themes and sub-themes. This ensured the confirmability of the data.

Second part: the intervention

A community-based quasi-experimental design was done with a sample size of 38 (who are different from the participants included in the qualitative study), with the following assumptions from a study by Astriani et al. (Mean score pre-test 2.0 (SD=0.5) and post-test mean score 2.7 (SD=0.3)) [22]. The power is set at 80%, and the alpha error is 5%. Adolescents were recruited using a convenience sampling procedure from the village of Gyabantha. From the total of 46 adolescents identified in the village through line listing, the required number of participants was contacted and recruited using a convenience sampling method. All adolescents aged 18-19 who consented to participate in the study were included; however, those undergoing treatment for severe mental health or substance use disorders were excluded.

The developed mind-map tool was implemented on the adolescents as demonstrations and educational sessions to improve the knowledge and attitude of the adolescents on mental health and substance abuse at the village Anganwadi Centre (AWC). The theoretical component included information, methods, and ways of addressing addiction and adverse outcomes, presented during a training session at the village AWC in the presence of the Accredited Social Health Activist (ASHA) and Anganwadi Worker (AWW) of the selected village. A total of four sessions were conducted, held once a week over a month, with each session lasting about 90 minutes. Supervision information was provided to the frontline workers to explain how they could monitor addiction and substance abuse among the participants. The frontline workers' queries and questions were addressed during the training sessions and through telephone conversations in the intervention phase. The follow-up period consisted of two visits by frontline workers on a random basis to each subject on different days to clarify any doubts they had. The participants were given a pre-test questionnaire in the form of a general socio-demographic proforma with the Patient Health Questionnaire-9 (PHQ-9) (mental health assessment) and the World Health Organization's (WHO) Alcohol, Smoking, and Substance Involvement Screening Test (ASSIST) (for substance abuse). Both questionnaires were already validated in the same setting. A post-test assessment of the participants, using the same PHQ-9 and ASSIST, was conducted 30 days after the completion of the intervention with the same participants. Scoring was done, and pre- and post-scores were compared.

The PHQ-9 tool used to screen for depression consists of nine questions. It is freely accessible for use [23,24]. In each question, the score ranges from 0 to 3. The total score ranges from 0 to 27. Scores of 1-4 are categorised as minimal depression, 5-9 as mild depression, 10-14 as moderate depression, 15-19 as moderately severe, and 20-27 as severe depression. The WHO-ASSIST questionnaire consisted of 10 questions for specific substance abuse. It is available as open access for use [25]. Scores of 0-3 suggest no intervention (except in alcohol 0-10), 4-26 require brief intervention, and more than 27 require intensive treatment. The questionnaire was administered by the investigator, ensuring the participants' privacy and confidentiality.

Ethical approval for the study was obtained from the All India Institute of Medical Sciences, Bhubaneswar (IEC number: T/IM-NF/CM&FM/23/149). Written informed consent was obtained from all participants aged 18 years or older, while written informed assent was obtained from participants under 18 years, along with parental consent.

For the quantitative component, all data were collected in a questionnaire, which was then entered into Microsoft Excel (Microsoft Corporation, Redmond, Washington). Statistical analysis was performed using the IBM SPSS Statistics for Windows, Version 22 (Released 2013; IBM Corp., Armonk, New York). Quantitative variables were expressed as means and standard deviations, while qualitative variables were analysed using frequencies and proportions. Correlations between quantitative variables were assessed with the Pearson correlation coefficient. A p-value of <0.05 was taken as statistically significant.

## Results

Qualitative results

A total of 18 participants' transcripts were included in the preparation of the mind map tool. The mean age of the participants was 17.5 (±2.3) years. The majority were females (77.8%), with males accounting for 22.2%. Most respondents (88.9%) were unmarried, while 11.1% were married. In terms of education, 83.3% were school- or college-going students, whereas 16.7% had dropped out. Regarding socio-economic status, two-thirds (66.7%) belonged to the upper-lower class, 22.2% to the lower class, and 11.1% to the middle class. Only one participant (5.6%) reported ever visiting an ARSH clinic, while the rest (94.4%) had never visited. Substance use or addiction was present in 16.7% of participants, whereas the majority (83.3%) reported no addiction. Mental illness was identified in 11.1% of the respondents, while 88.9% did not report any such condition. Suicidal ideation was reported by one participant (5.6%). When asked about coping mechanisms for stress and emotions, leisure activities such as listening to music or watching television were the most common (66.6%), followed by playing (16.7%) and withdrawal from social interaction (16.7%). All participants reported being physically active (Table 1).

**Table 1:** Socio-demographic characteristics of the participants included in the mind map tool development (n=18)

Variables	n (%)
Age (mean (SD))	17.5 (2.3)
Sex	
Male	4 (22.2)
Female	14 (77.8)
Marital status	
Unmarried	16 (88.9)
Married	2 (11.1)
Education	
School/college-going	15 (83.3)
Drop-out	3 (16.7)
Socio-economic status	
Lower	4 (22.2)
Upper lower	12 (66.7)
Middle	2 (11.1)
Visit to ARSH clinic	
Visited	1 (5.6)
Not visited	17 (94.4)
Addiction (self-reported)	
Present (current user)	3 (16.7)
Absent (not used in the last one month)	15 (83.3)
Presence of mental illness (self-reported)	
Present	2 (11.1)
Absent	16 (88.9)
Thought of committing suicide	
Yes	1 (5.6)
No	17 (94.4)
Most common coping mechanism to deal with stress & emotion	
Playing	3 (16.7)
Leisure activities	12 (66.6)
Stop talking to others	3 (16.7)
Physically active (self-reported)	
Yes	18 (100.0)
No	0 (0.0)

Substance abuse and mental health challenges arise from various factors, including academic stress, peer pressure, family issues, and relationship problems. Students often experience anxiety, fear of exams, and career stress, which can lead to excessive anger, lack of self-confidence, and difficulties in managing time. Peer influences, including negative behaviour, peer pressure, and provocation, significantly shape individual outcomes. Family-related challenges such as financial strain and occupational stress further add to the burden. In addition, relationship difficulties like breakups, along with health-related concerns, often lead to physical and psychological problems, ranging from mood swings, sleep disturbances, and eating disorders to memory impairment, and in severe cases, psychosis or even suicide. Treatment options range from pharmacotherapy, such as medical consultations, medications, and interventions in de-addiction centres, to non-pharmacological therapies like counselling, psychotherapy, and coping mechanisms, including engaging in recreational activities like singing, dancing, or travelling. Emotional support from family and friends is crucial, although individuals may still experience feelings of loneliness and struggle to share their difficulties (Table 2).

**Table 2:** Themes, domains, and codes

Themes	Domains	Codes
Reasons for substance use/mental health challenges	Study issues	Anxiety, fear of exams, stress of career, excessive anger, stress of school, loss of hope, lack of self-confidence, problem in time management, unsatisfactory marks in the exam
	Peer influence	Bad behaviour of friends, peer pressure, provocation of friends
	Family Issues	Family issues, financial hardship, work-related issues
	Relationship challenges	Relationship problem, break up with a friend, unawareness
Health consequences	Physical health consequences	Mood swings, impact on physical health, diseases, decreased sleep, decreased appetite, Eating disorders
	Psychological health consequences	Dependence, memory loss, withdrawal symptoms, psychosis, suicide
Treatment options	Pharmacotherapy	De-addiction centre, medical consultation, medicine consulting, local healer
	Non-pharmacological therapy	Diversional therapy, counselling, decreasing craving, quitting the substance, psychotherapy
Coping mechanism	Recreational activities	Singing, dancing, drawing, playing a hobby, travelling/going out
	Emotional support	Discussing with family, counselling by friends, emotional support, motivation
	Loneliness	Not able to share solitude

Reasons for substance abuse and mental health issues in adolescents

The most common reasons for substance abuse and mental health problems of adolescents are stress associated with exams, study, marks, or career opportunities in the present study. However, family issues like internal conflict, conflict with parents, and financial hardship also play a crucial role. Sometimes adolescents get influenced by bad company or peer groups. In the modern era, relationship failure also affects the mental health of young minds and influences the use of various harmful addictive substances.

According to P1 (P = Participant identifiers), "Many peer groups will use the substance, and they may get affected by it. If friends are calling, then they are not able to deny. They are getting the desire of smoking and drinking and they are doing it. They think that they will not be doing it the next time. But once it gets started, they will have the desire to do it again and again."

P4 reported that "They can get affected by relationship issues and family problems. Sometimes they are not able to score good marks in the exam. They think themselves that they will not be able to do anything in their lives. Then they may start using various substances."

Consequences on the health of the adolescents

It can affect not only the physical health but also the psychological health of young adults. Adolescents reported being aware of cases where substance abuse has led to damage to kidneys, livers, and other organs. It can cause mood swings and decreased sleep. Substance abuse may cause physical dependence or withdrawal reactions. Various mental health issues, like depression or anxiety disorders, can cause psychosis and even snatch young lives by committing suicide.

P2: "It is impacting the physical health. Many people are not aware of it. It can affect the kidneys, liver, and other organs. It can affect all parts of the body. Even if it causes dependence, although you want to stop, you can’t quit it easily."

P6: "Depression, anxiety, and hallucination are different types of mental disorders in adolescents. Many adolescents are now committing suicide due to various mental health issues."

Treatment of substance abuse and mental health problems

They can seek pharmacotherapy, i.e., visit any hospital and do a doctor's consultation. On the other hand, they can receive counselling or psychotherapy. Adolescents with substance abuse can also visit a de-addiction centre for treatment. However, constant support from the family or friends is required to quit substance abuse or come out of a stressful period in life.

P3: "Medicine can be taken. Counselling can be beneficial for some. They can go to the hospital to consult a doctor. The person has to be self-determined. The person has to be motivated. The family support can be given."

P5: "I don’t know much about it. We will do counselling. If someone is not willing, they can't stop it. We will bring them to medical and will give them medicines and good food."

P8: "We can bring them to the doctor. Take him or her for an outing, which may refresh the mind. They can also go shopping. Family members and friends can counsel them."

Coping mechanisms of the adolescents

Recreational activities, such as singing, dancing, playing, and drawing, were described by the adolescents as the most common coping mechanisms. Many of them even prefer to travel to escape the monotonous life. Emotional support from family and friends creates a supportive environment for young minds. However, a few of them mentioned preferring solitude or loneliness during the stressful episode.

P4: "Dancing or singing, anything can be done to keep the mind free. Family should encourage to do the things. But they should not tell you you will not be able to do it. Whenever I am making a painting, someone appreciating it will give me support to do it better next time."

P7: "Good children. Family always supports. Those who don't listen to their parents, none of the parents support them. Family can give emotional support. They can make them understand not to have misunderstandings. Not to have depression."

Quantitative results

Most participants were female (24 out of 38). Only two of them were married. The majority of the participants were continuing their education, while only eight had dropped out of school. The majority are of upper-lower socio-economic status. Only one participant had previously visited the ARSH clinic and received counselling. Three participants were current tobacco users. Two participants were reported to have mental health illnesses, but none of them were on any medication. One of them had reported their thought of committing suicide. Most participants preferred leisure activities, such as watching TV, playing, singing, or dancing, as a modality to reduce stress during their difficult time. Only three of the total 38 participants reported a preference for solitude and stopping talking with others after experiencing any emotional or mental stress. They do not prefer any support during episodes of stress. However, 15 participants preferred to seek help from family or friends (Table 3).

**Table 3:** Socio-demographic characteristics of the participants included in the intervention (n=38) ARSH = Adolescent Reproductive and Sexual Health

Variables	n (%)
Sex	
Male	14 (36.8)
Female	24 (63.2)
Marital status	
Unmarried	36 (94.7)
Married	2 (5.3)
Education status	
Continuing education	30 (78.9)
School drop-out	8 (21.1)
Socio-economic status	
Lower	8 (21.0)
Upper-lower	23 (60.5)
Middle	7 (18.5)
Visit to ARSH clinic	
Yes	1 (2.6)
No	37 (97.4)
Tobacco use	
Yes	3 (7.9)
No	35 (92.1)
Mental illness	
Present	2 (5.3)
Absent	36 (94.7)
Suicidal ideation	
Yes	1 (2.6)
No	37 (97.4)

The pre-test mean score of the PHQ-9 among the participants was 7.06 (SD=3.38). However, it was reduced to 5.78 (SD=2.50) after the intervention (Table 4).

**Table 4:** PHQ-9 and WHO ASSIST scores before and after the mind map intervention (n=38) PHQ-9 = Patient Health Questionnaire-9; WHO ASSIST = World Health Organization's (WHO) Alcohol, Smoking, and Substance Involvement Screening Test

Questionnaire	Pre-test Mean (SD)	Post-test Mean (SD)	p-value
PHQ-9	7.06 (3.38)	5.78 (2.51)	<0.001
ASSIST	2.89 (4.62)	1.83 (3.55)	<0.001

**Figure 5:**
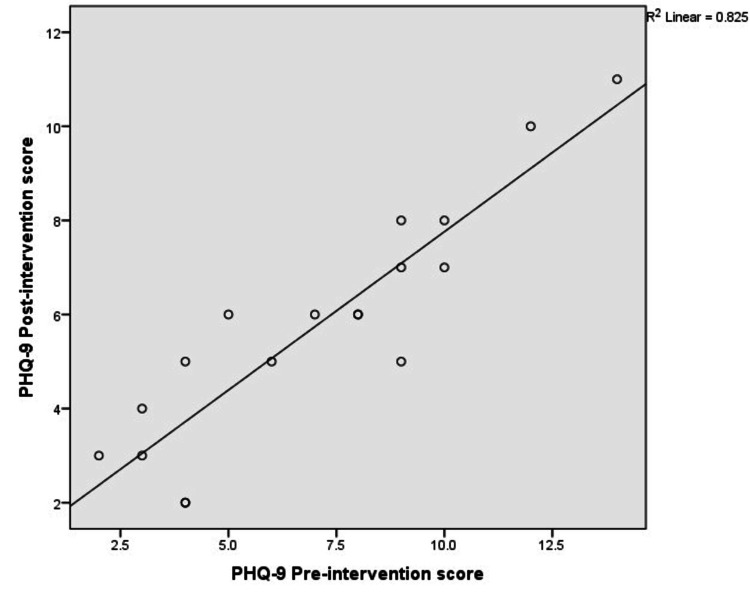
Correlation between pre- and post-scores of PHQ-9 Pearson correlation significance <0.001. PHQ-9 = Patient Health Questionnaire-9

**Figure 6:**
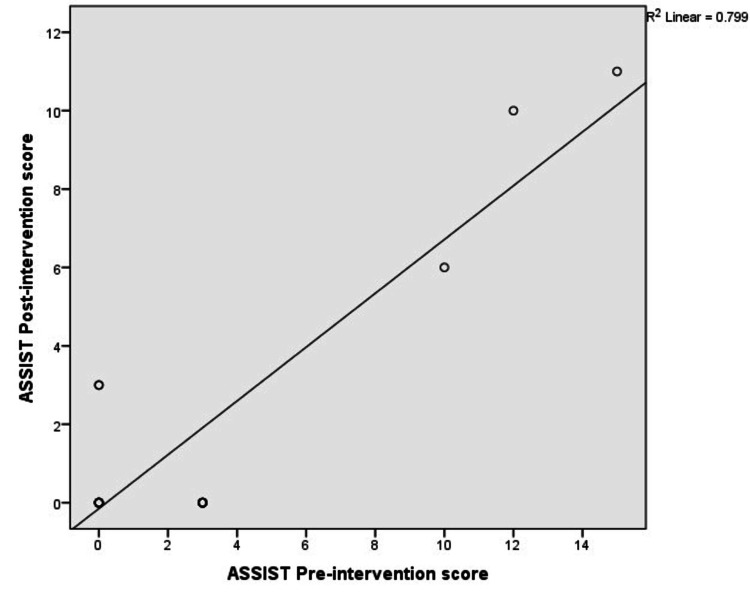
Correlation between pre- and post-scores of the WHO ASSIST tool Pearson correlation significance <0.001. WHO ASSIST = World Health Organization's (WHO) Alcohol, Smoking, and Substance Involvement Screening Test

We found a significant correlation between the pre- and post-test PHQ-9 scores (p < 0.001, r² = 0.82), suggestive of a high correlation. A similar finding was reported in the WHO ASSIST tool, where the score was reduced from 2.89 (SD=4.62) to 1.83 (SD=3.80), with a significant correlation (p<0.001, r²=0.79, suggestive of a high correlation) (Figures 5, 6).

## Discussion

Substance abuse and mental health disorders are one of the biggest threats to adolescents across the globe. Unhealthy behaviours frequently develop during adolescence, posing significant public health challenges. Substance abuse, in particular, profoundly affects individuals, families, and communities. Its cumulative effects lead to costly social, physical, and mental health issues. Several interventions have been implemented for adolescents to reduce substance abuse, including non-pharmacological approaches such as brief counselling, mindfulness interventions, and cognitive behavioural therapy, as well as various pharmacological treatments [11]. Although there are several promising treatments, psychosocial interventions still remain the primary modality of treatment. A systematic review and meta-analysis by Steele et al. found that motivational interviewing and psychoeducation were effective in reducing substance abuse disorder among adolescents [20].

While drug use and addiction can occur at any stage of life, it often begins during adolescence, a time when the initial signs of mental illness also tend to emerge. Many mental health disorders begin to emerge in late childhood and early adolescence, adding to the burden these conditions impose on young people and continuing to affect them later in life [6]. Adolescent coping skills and resilience curricula showed improvement in depressive symptoms, cognitive abilities, academic stress, problem-solving, and overall mental well-being [6]. A meta-analysis by Zheng et al. had found that interpersonal therapy can be used as an effective tool to reduce depressive symptoms among adolescents [26]. Focusing on integrated digital, community, and school platforms within child and adolescent health services could greatly benefit from increased emphasis on early recognition and prevention of mental health issues.

Mind maps have emerged as a valuable tool for visualising complex information, fostering critical thinking, and enhancing the organisation and interpretation of concepts [17]. In this study, the mind mapping tool was employed to address adolescent mental health and substance abuse issues, with the objective of improving their behavioural outcomes. The paired t-test revealed a statistically significant difference between the pre- and post-intervention scores on the PHQ-9 and WHO ASSIST tools, indicating the effectiveness of this approach. A similar correlation between addiction and depression has been found in a systematic review [27]. Substance use and mental health conditions often co-occur, with depressive disorders being one of the most frequently associated psychiatric complications. One study reported that more than 25% of patients experienced a substance-induced depressive episode in their lifetime [28]. The reduction in both depressive symptoms and substance use behaviours suggests that mind maps can be a powerful intervention in this context.

Previous research supports the utility of mind maps in educational and cognitive development settings. For instance, a study conducted in Istanbul demonstrated that mind mapping effectively promoted critical thinking and idea generation among children [16]. These findings align with our study, in which mind mapping contributed to improved mental health outcomes by helping adolescents understand and manage their conditions.

Substance abuse during adolescence disrupts the development of the prefrontal cortex, hippocampus, and limbic circuits, altering the brain's reward pathway (VTA-nucleus accumbens) and impairing decision-making and impulse control [9]. The impact of substance abuse on adolescent brain development is well-documented, and interventions that enhance cognitive functions, such as mind mapping, may mitigate some of the adverse effects associated with substance abuse [17]. Our study's results are consistent with findings from Indonesia, where mind mapping has been shown to significantly improve self-awareness related to smoking cessation [18]. This highlights the potential of mind mapping not only as an educational tool but also as a method of behavioural change.

Moreover, our study identified stress, peer influence, and family issues as significant factors contributing to substance abuse and mental health problems among adolescents. These results are consistent with findings from a school-based study in the USA, which emphasised that increased awareness, community understanding, and appropriate interventions could help prevent adolescent substance abuse [19]. Additionally, research conducted by Maya et al. (2020) in Goa and Delhi demonstrated that problem-solving, goal setting, and psychoeducation were effective in reducing anxiety symptoms among adolescents [29]. This further supports the notion that structured interventions like mind mapping can play a crucial role in addressing mental health challenges.

Provision of accessible and community-acceptable psychiatric services for adolescents is the need of the hour, but there are still financial and service structure barriers to be overcome to achieve the objectives outlined here [30]. School-based primary prevention programmes have proven effective in addressing combined substance abuse. However, the effectiveness of internet-based interventions, policy initiatives, and incentive-based approaches remains inconclusive, indicating a need for further research to better understand their impact [6].

Overall, our study illustrates that mind mapping is a promising tool for enhancing adolescent mental health and managing substance abuse. The significant improvements in PHQ-9 and WHO ASSIST scores following the intervention suggest that mind mapping can effectively support cognitive and behavioural changes in this population. Future research should explore the long-term impacts of such interventions and their potential for broader application in diverse settings. This study employs qualitative designs to map the problem, providing a comprehensive understanding of the behaviour.

Some of the limitations of the study are the smaller sample size in the planning and intervention phase, which makes it challenging to generalise results; single-village convenience sampling, which limits generalisability; the lack of assessor blinding, which introduces potential measurement bias; and the 30-day follow-up, which is insufficient to assess sustained behavioural change in substance abuse. Mind mapping as an intervention tool has limited utility for new or established addictive behaviours. As it is a quasi-experimental design without a comparator, the outcome of the study could be better supported by higher study designs. The lower follow-up period after intervention is another challenge of this study.

## Conclusions

The study demonstrated that mind mapping is an effective tool for improving adolescent mental health and reducing substance abuse in rural Odisha. The significant reduction in PHQ-9 and WHO ASSIST scores highlights its potential for enhancing decision-making and awareness among adolescents. To build on these positive results, it is recommended that mind mapping interventions be scaled up across other rural communities. Additionally, integrating this approach with school-based programmes and family counselling could further strengthen its impact. Continued monitoring and support from local health systems and community-based organisations will be critical for long-term sustainability.
